# Iron Overload: Pathophysiology, Diagnosis and Monitoring

**DOI:** 10.1111/ijlh.70132

**Published:** 2026-05-04

**Authors:** Elena Chatzikalil, Polyxeni Delaporta, Konstantinos Bistas, Antonis Kattamis

**Affiliations:** ^1^ Thalassemia Unit, First Department of Pediatrics National and Kapodistrian University of Athens Athens Greece; ^2^ ‘Aghia Sophia’ Children's Hospital ERN‐EuroBloodNet Center Athens Greece

**Keywords:** ferritin, hemochromatosis, iron overload, thalassemia, transferrin saturation

## Abstract

Iron overload is associated with significant health risks, underscoring the importance of understanding its pathophysiology as well as establishing accurate diagnostic and monitoring methods. Chronic iron overload is associated with either genetic disorders characterized by excessive iron accumulation (hereditary hemochromatosis), or is secondary to diseases of ineffective erythropoiesis and/or requiring regular blood transfusions (like thalassemia, sickle cell disease, myelodysplastic syndromes). Diagnosis is based on clinical suspicion, corroborated by laboratory findings such as elevated serum ferritin and transferrin saturation, along with imaging techniques (magnetic resonance imaging) which facilitate non‐invasive assessment of iron levels in parenchymal organs. Elevated ferritin and transferrin saturation and exclusion of secondary causes should prompt genetic evaluation for hereditary disorders predisposing iron overload. Chronic systemic iron overload causes progressive tissue iron accumulation, leading to severe clinical implications, including myocardial dysfunction, liver cirrhosis, and increased risk of hepatocellular carcinoma. Monitoring includes evaluating iron overload indices (serum ferritin, transferrin saturation, liver and heart iron concentration) along with serum and urine indices of parenchymal organ damage at different timepoints regarding the type of disease, patient's age, severity and response to treatment, and aims in improving disease progression and preventing complications. This article provides a comprehensive overview of the pathophysiologic mechanisms and the diagnostic and monitoring techniques of iron overload, in order to revise current knowledge and to raise clinical awareness for effective management.

## 1 Introduction

Iron overload occurs when iron levels exceed physiological requirements due to either genetic causes (e.g., hereditary hemochromatosis), increased iron absorption, or secondary to transfusions of red blood cells (e.g., transfusion dependent anemias) [1, 2]. Impaired iron homeostasis may lead to increased intestinal iron absorption and iron release from macrophages, further resulting in elevated transferrin saturation (TSAT) and an expanded circulating iron pool [1]. TSAT is a key biomarker for measuring iron availability, with levels above 45% indicating iron overload and levels exceeding 60% to 70% being associated with free iron generation, which can be detrimental to hepatic parenchymal cells [3]. Hyperferritinemia is defined by serum ferritin levels above the upper reference ranges, which vary with age, gender and laboratory methods [4]. Upper cut‐off is typically set to 200 μg/L for females and 300 μg/L for males [5]. High ferritin alone does not indicate iron overload [5]. Elevated TSAT and serum ferritin, and exclusion of causes of secondary hemosiderosis, should prompt evaluation for genetic causes of iron overload [6]. Further evaluation of the severity of iron overload can be achieved with the use of MRI. In this respect, non‐invasive hepatic iron qualification using MRI techniques is recommended, as systemic iron overload is typically associated with increased liver iron concentration [7].

Hemochromatosis, the main cause of genetically originated iron overload, is an inherited autosomal recessive disease, with approximately 80% of affected European individuals carrying the homozygous *C282Y* alteration in the *HFE* gene [7]. In rare cases, hemochromatosis is caused by recessive pathogenic genetic alterations in genes encoding either the hormone hepcidin (*HAMP*), transferrin receptor 2 (*TFR2*), or hemojuvelin, as well as gain of function dominant variants in the gene encoding ferroportin (*SLC40A1*) [8]. The pathophysiology of iron overload diseases has greatly advanced due to significant improvements in our understanding of iron metabolism in the different forms of the disease. The different types of hereditary hemochromatosis are characterized by a deficiency in the function of hepcidin, which is the hormone that negatively regulates plasma iron levels. Thus, increased plasma iron leads to the circulation of iron in a non‐transferrin‐bound form, which can accumulate in parenchymal cells and lead to systemic hemosiderosis [9]. Ferroportin disease demonstrates a different mechanism of iron overload, involving defective transport of cellular iron into the plasma and leading to iron deposition primarily within the spleen macrophages [10]. Acquired iron overload is commonly observed in hemoglobinopathies and myelodysplastic syndromes and is associated with ineffective erythropoiesis, being characterized by misappropriately decreased hepcidin levels relative to iron levels, and/or iatrogenic due to multiple blood transfusions [11].

Management of systemic iron overload depends on the severity of organ involvement and liver disease stage [7]. Timely institution of therapeutic phlebotomy and/or chelation treatment can effectively prevent organ damage and even reverse iron‐induced toxicity in parenchymal cells [7]. Early diagnosis and appropriate monitoring are vital for preventing iron overload‐related complications. The aim of this article is to provide a detailed overview of the pathophysiology of iron overload, and to analyze currently used diagnostic and monitoring methods.

## 2 Materials and Methods

An in‐depth review of the available literature was undertaken using Google Scholar and keyword searches including [Iron overload] in combination with [ferritin], [TSAT], [hemochromatosis], and [thalassemia], limiting the search to English language articles. All articles were reviewed for relevance and collated by the author.

## 3 Results

### 3.1 Pathophysiology

Iron is a highly reactive molecule, which easily alternates between ferrous (Fe^2+^) and ferric (Fe^3+^) states resulting in the gain and loss of electrons [1]. This capability is essential for maintaining cytochromes' function, DNA synthesis, cellular metabolism, and growth, while at the same time, it leads to harmful free radical generation, damaging cell membranes, proteins, and nucleic acids, and leading to fibrotic changes and cell death [12]. Iron enters the human body through two main routes: via the placenta during fetal development and through the small intestine postnatally [1, 13]. Daily dietary iron intake is 10–20 mg, with 1–2 mg absorbed in the proximal duodenum [1]. Iron absorption is mainly regulated by the expression of divalent metal transporter‐1 (DMT‐1) and a ferrous reductase which converts ferric iron to its ferrous form, through enterocytes in the duodenum and proximal jejunum [1]. Once absorbed, iron can be stored as ferritin within enterocytes or exported into the bloodstream via ferroportin, the only iron‐exporting protein [1]. The liver‐derived protein transferrin binds and transports two Fe^3+^ ions in the plasma, safely delivering toxic iron to tissues where it can be stored bound to ferritin [14]. Hepcidin, which is secreted by the liver, plays a vital role in this regulation by binding to ferroportin, promoting its degradation and thereby controlling iron release into circulation mainly from reticuloendothelial cells and enterocytes [15]. Intestinal crypt cells adjust iron absorption by regulating DMT1 and ferroportin [16]. Intracellular iron uptake is regulated by the IRP system and influenced by cellular iron levels and oxygen availability [17].

Total body iron levels typically range from 3 to 5 g, with approximately 60% utilized in hemoglobin and 10% in myoglobin, and the remaining iron being stored in the liver and macrophages [18]. Approximately 1–2 mg is lost daily through sweat, blood loss, and other means. To balance this loss, the body needs to absorb a similar amount from the diet [18]. Hemoglobin production requires 20–25 mg of iron daily, necessitating tight regulation and recycling [18].

**FIGURE 1:**
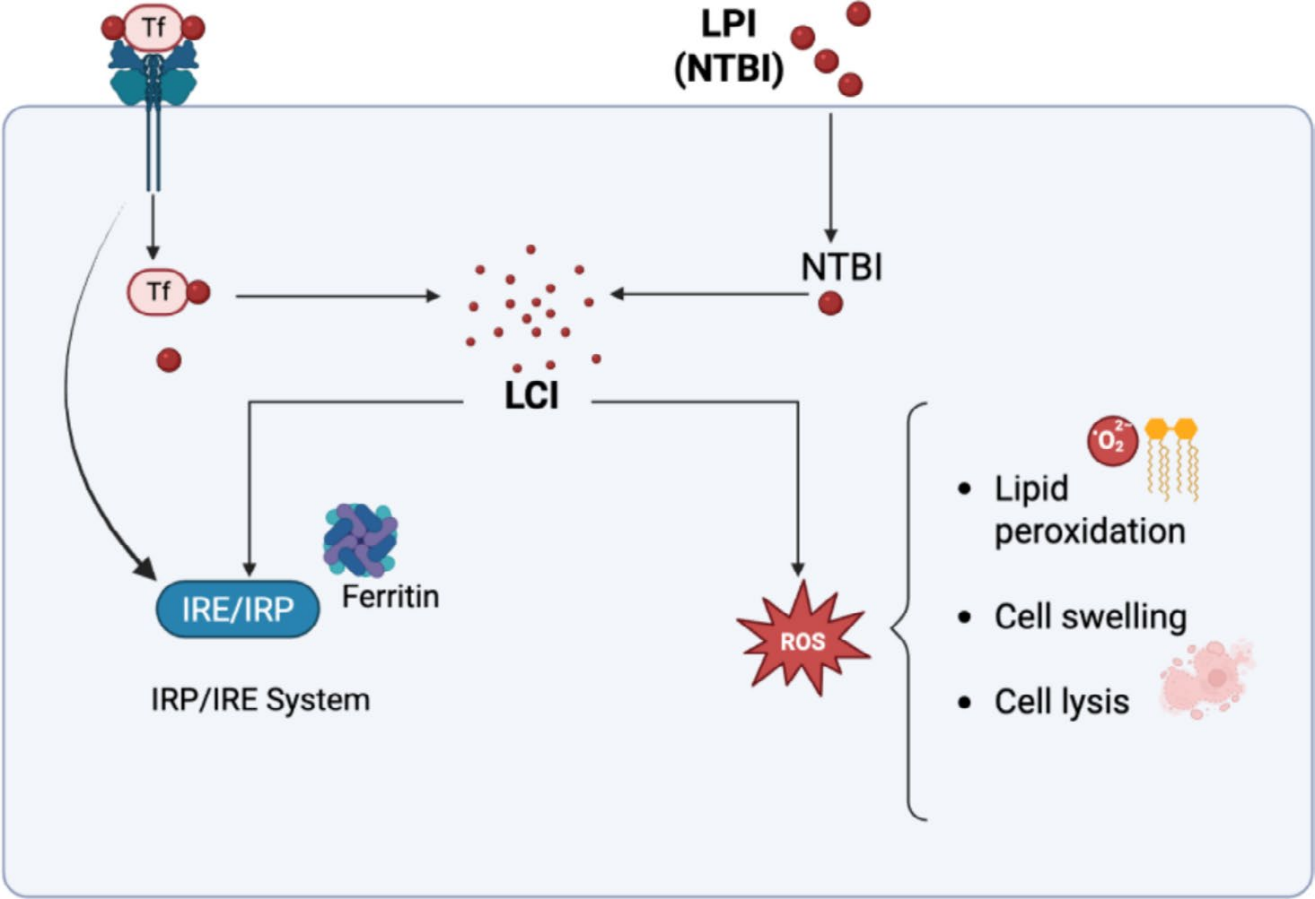
Transferrin bound iron complex enters the cell by binding to transferrin receptor and is stored in the form of ferritin via IRP/IRE system. NTBI iron enters the cells via unregulated pathways and contributes to the formation of labile cell iron pool. Excessive iron from the labile cell iron pool leads to ROS formation, further resulting in peroxidation of the cell membranes, cell swelling and cell lysis. LCI, Labile cell iron; NTBI, non‐transferrin‐bound iron; TF, transferrin. The figure was created using BioRender software version 04, License # LY2960RB8P.

Systemic iron overload is characterized by elevated plasma iron levels and by iron accumulation in parenchymal cells [18]. In conditions of iron overload, the iron binding sites of transferrin become saturated and iron species that are not bound to transferrin are present in plasma [19]. Eventually, if transferrin saturation exceeds 75%, a form of low molecular non‐transferrin‐bound iron is produced, called labile plasma iron or plasma non‐transferrin bound iron (NTBI) [19]. The appearance of NTBI, which presents a high propensity for redox activities, producing highly reactive oxygen species (ROS), is the primary pathogenic event of toxicity induced by iron overload [20]. The NTBI intracellular uptake is fundamentally different from the controlled pathway of iron uptake through the transferrin/transferrin receptor coupling, and is thought to involve voltage‐gated calcium channels (VDCC) and the Zn^2+^ transporter ZIP14 [21]. Enzymic and non‐enzymic antioxidants maintain ROS within a narrow physiologic range by disposing excess ROS and ferritin [22]. Significant intracellular iron overload leads to chronic and uncontrolled increase of ROS resulting in unbalanced oxidative stress. The latter gradually results in lipid peroxidation of cell membranes, peroxidative damage of inner membrane phospholipids, dysfunction in the transport of solutes and ions with hydroperoxide‐induced release of Ca^2+^ due to opening of Ca^2+^ pore in inner membranes, and finally, cell swelling and lysis [23] (Figure 1).

In Western populations, persistent iron overload, in absence of an underlying cause, is mostly attributed to genetic diseases, mainly hereditary hemochromatosis, caused by genetic alterations in genes involved in the sensing of systemic iron levels (*HFE*, *HJV*, *HAMP*, and *TFR2*), other genetic causes of primary iron overload (hereditary aceruloplasminemia, ferroportin disease, and hereditary atransferrinemia), or to chronic disorders that cause ineffective erythropoiesis and secondary iron overload (like thalassemia, sickle cell disease, myelodysplastic syndromes) [7, 20]. Chronic iron overload affects parenchymal organs, including the liver, heart, endocrine glands, and pancreas, reflecting the pattern of tissue iron uptake from NTBI [18]. Not all tissues take up NTBI at the same pace; some tissues (e.g., skeletal muscle) are spared from iron loading while others (myocardial muscle, endocrine tissue, and hepatocytes) are susceptible to the toxic effect of excess iron [18]. Hepatic iron overload may lead to fibrotic changes and eventually cirrhosis and hepatocellular carcinoma [24]. Myocardial iron overload may induce cardiomyopathy and heart failure [25]. Chronic hemosiderosis also may lead to pituitary damage, resulting in hypogonadism, growth retardation, and delayed puberty, and to other endocrine complications like diabetes mellitus, hypothyroidism, and hypoparathyroidism [7, 26].

**FIGURE 2:**
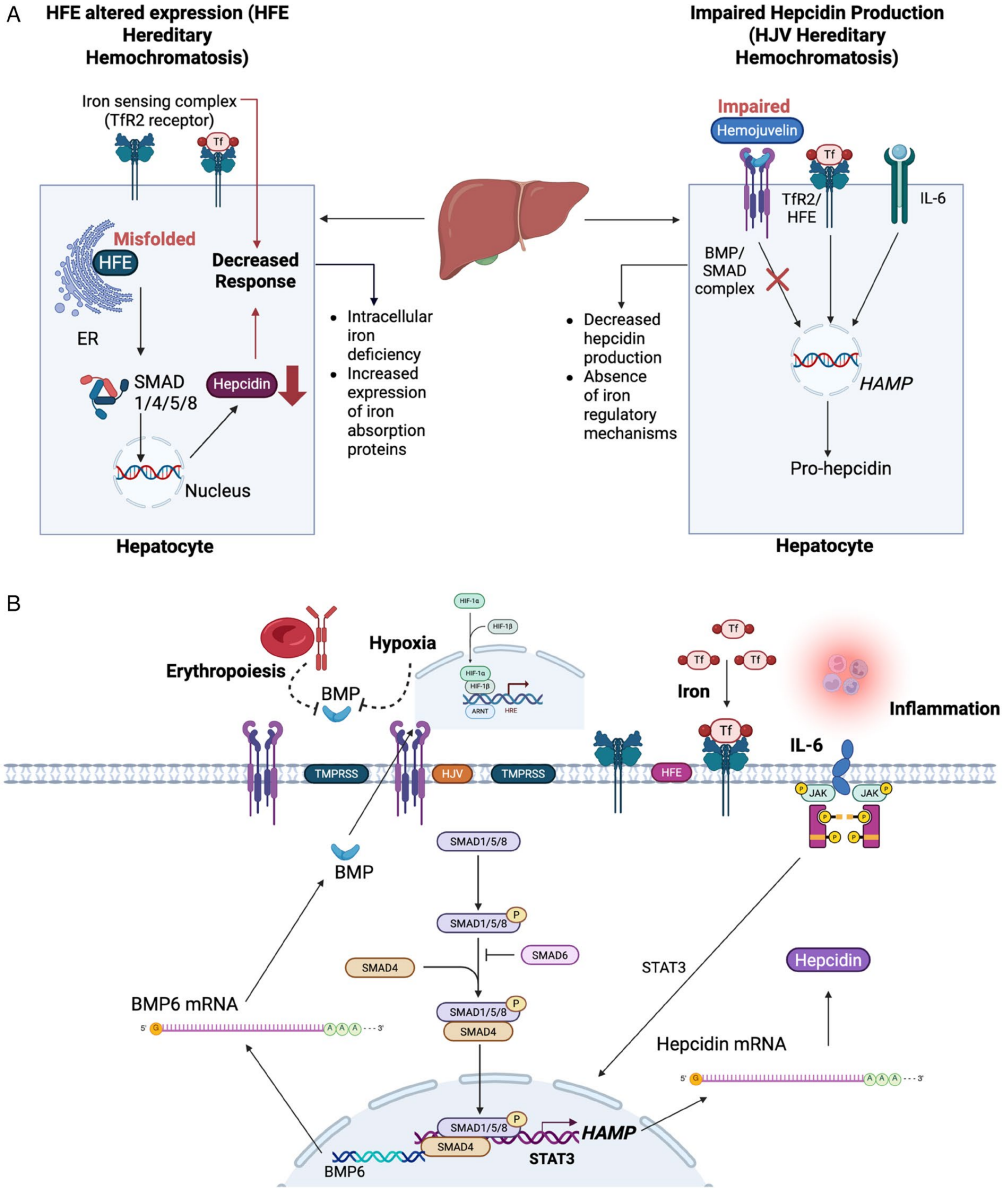
(A) Presentation of the main mechanisms of inherited iron overload. In HFE hemochromatosis, mutated HFE proteins are trapped inside cells, leading to “intracellular iron deficiency” and increased expression of iron absorption proteins, resulting in excess iron absorption. Iron sensing complex and hepcidin production are dysregulated. Mutations in HJV (hemojuvelin hemochromatosis) disrupt BMP‐SMAD signaling, leading to hepcidin deficiency, and excessive iron release into the bloodstream. BMP, bone morphogenic protein; ER, endoplasmic reticulum; IL‐6, interleukin 6; SMAD, mothers against decapentaplegic; TfR1/2, transferrin receptor. (B) Increased systemic iron levels in diseases of ineffective erythropoiesis decrease hepcidin expression. Saturation of TFR1 leads to the binding of BMP6 receptors on the cell membrane. Increased liver iron concentration stimulates the expression of BMP6 genes, leading to increased production of BMP6 proteins that interact with BMP receptors. BMP6 binding to these receptors recruit SMAD proteins, and the phosphorylation of the SMAD1/5/8‐SMAD4 complex allows BMP6 translocation to the nucleus, activating hepcidin expression. Increased erythropoiesis and hypoxic activity result in the secretion of ERFE from erythroid precursors cells, dampening the BMP6 signaling pathway. Moreover, inflammatory JAK–STAT pathway activation promotes hepcidin expression independently of iron status and erythropoietic activity. BMP6, bone morphogenic protein 6; HAMP, hepcidin antimicrobial peptide; HIF, hypoxia‐inducible factor; IL‐6, interleukin 6; JAK–STAT, Janus kinase/signal transducer and activator of transcription; TMPRSS6, transmembrane serine protease 6. The figure was created using BioRender software version 04, License #TJ2961M6R2.

The mechanism of excess iron accumulation differs regarding the underlying cause (Figure 2).

HFE, which is altered in the majority of hereditary iron overload cases, is a transmembrane protein crucial for iron uptake into macrophages and intestinal epithelial cells [27]. Normal iron absorption involves HFE interacting with β2‐microglobulin and transferrin receptor (TfR‐1) to enhance transferrin‐bound iron entry into cells (endotheliosis) [16, 28]. Mutated HFE proteins are trapped inside cells, leading to “intracellular iron deficiency” and increased expression of iron absorption proteins, resulting in excess iron absorption, which exceeds normal dietary levels [29]. Chronically, this condition leads to excess iron deposits in parenchymal tissues (heart, liver, pancreas) [29]. Hepcidin is the major regulator of iron homeostasis; it inhibits intestinal iron absorption and release from macrophages by binding to ferroportin [30]. A key pathological feature of hemochromatosis is insufficient (or absent in *HJV* hemochromatosis) hepcidin production [30]. The complexity of hepcidin regulation partially explains the absence of disease modifiers, although co‐factors like alcohol or viral hepatitis may directly suppress hepcidin production worsening deficiency [31]. Regarding non‐hemochromatosis inherited disorders of iron overload (ferroportin disease), mechanisms include either ferroportin loss of function, which results in iron accumulation in macrophages and enterocytes, leading to high serum ferritin but low to normal transferrin‐iron saturation, or hepcidin resistance, which mimics hepcidin deficiency, resulting in increased iron absorption, high TSAT, and iron deposition in the liver [29].

Secondary iron overload is mainly encountered in diseases which require frequent blood transfusions and in diseases of ineffective erythropoiesis. In transfusion dependent β‐thalassemia, which is a disease‐model of secondary hemosiderosis, frequent blood transfusions lead to iron accumulation in parenchymal organs causing toxicity, as there is no natural way to eliminate excess iron [32]. Secondary iron overload related to ineffective erythropoiesis is associated with more complex mechanisms. Ineffective erythropoiesis suppresses hepcidin synthesis, resulting in increased iron absorption from the gut, despite regulatory feedback from iron stores [33]. Factors like Growth Differentiation Factor 15 (GDF‐15) and other cytokines suppress hepcidin, perpetuating iron overload. EPO‐induced ERFE affects hepcidin through the BMP6 pathway [33]. BMP6 aids in sensing iron, but its regulation is impaired by ineffective erythropoiesis, contributing significantly to iron overload [34].

## 4 Diagnosis

Iron load is commonly estimated by ferritin levels. Hereditary and acquired causes of iron overload are presented in Table 1. Falsely elevated ferritin levels are observed in many inflammatory conditions, limiting the specificity of a single measurement to ascertain iron overload. One of the earliest biochemical abnormalities indicating iron overload is an elevated TSAT. A TSAT over 45% can identify most hemochromatosis homozygotes, while a TSAT below 45% typically rules out hereditary hemochromatosis and, in cases of high serum ferritin levels (above 300 μg/L in males and above 200 μg/L in females), is an indicator of other causes, mainly hematological disorders (thalassemia, sickle cell disease, myelodysplastic syndromes), alcohol‐related liver disease, metabolic‐dysfunction associated liver disease (MASLD), viral hepatitis, acute liver injury, chronic inflammation, and metabolic syndrome [7]. In cases of persistently elevated serum ferritin levels (above 500 μg/L) and normal TSAT, inherited diseases of iron overload, other than hemochromatosis (hyperferritinemia‐cataract syndrome, ferroportin disease, aceruloplasminemia, benign hyperferritinemia without cataracts), should be considered and diagnosed via genetic sequencing after clinical suspicion and exclusion of other secondary causes [7]. For family screening, it is recommended that first‐degree relatives of patients with genetically confirmed hemochromatosis undergo genotyping, as penetrance is considered higher in family members compared to the general population [35].

**TABLE 1:** Inherited and acquired causes of iron overload.

Inherited iron overload	Acquired iron overload
Hereditary hemochromatosis: *Type 1*: *HFE* (6p21.3) genetic alterations *Type 2*: A: *HJV* (1q21) genetic alterations, B: *HAMP* (19q13) genetic alterations *Type 3*: *TFR2* (7q22) genetic alterations *Type 4*: *SLC40A1* (2q32) genetic alterations	Diseases of ineffective erythropoiesis: thalassemia, sideroblastic anemia, myelodysplastic syndromes
Aceruloplasminemia: *CP* (3q23‐24) genetic alterations	Recurrent blood transfusions (e.g., transfusion dependent b‐thalassemia)
Atransferrinemia: *TF* (3q22) genetic alterations	Liver disease: alcoholic liver disease, viral hepatits
Hereditary benign hyperferritinemia (H‐ gene genetic alterations)	Inflammation
*DMT1* genetic alterations	Other (e.g., porphyria)

Recent advances in understanding the pathophysiology and molecular basis of iron metabolism have revealed that hereditary hemochromatosis is caused by mutations in at least five genes, leading to insufficient hepcidin production or, in rarer cases, resistance to its action [8]. This has resulted in a revised hemochromatosis classification, based on various molecular subtypes (Table 2) [8]. In clinical settings, type 1 hemochromatosis genes (*HFE*) genotyping should follow the assessment of serum iron parameters and is mainly performed on individuals demonstrating biochemical evidence of iron overload. While there is no universally accepted threshold for ferritin or TSAT levels, it is suggested to proceed to *HFE* genotyping in individuals with TSAT above 45% and ferritin above 300 μg/L in males and above 200 μg/L in females [7]. In a large cross‐sectional study, 85% of men and 73% of women homozygous for the HFE p.C282Y variant demonstrated elevated TSAT, while ferritin levels exceeded 300 μg/L in 88% of homozygous males and 200 μg/L in 57% of homozygous females [36]. *HFE* gene has three common mutations: *C282Y, H63D*, and *S65C* [37]. Only the *C282Y* homozygous mutation is clearly associated with hereditary hemochromatosis, while *H63D* or *S65C* mutations do not cause significant iron overload in the absence of additional factors (e.g., alcohol, hepatitis C) [37]. EASL guidelines recommend HFE genotyping for each European shows biochemical evidence of unexplained iron overload [7]. Disease penetrance of HFE‐hemochromatosis is affected by both sex and age (increasing with increasing age) [36].

**TABLE 2:** Hemochromatosis new classification.

Disease	Type	Gene	Protein	Inheritance	Phenotype	OMIM#
HFE‐related HH	1	*HFE*	HFE	AR	Classic	#235200
Non‐HFE‐related HH	2a	*HJV*	Hemojuvelin	AR	Juvenile	#602390
Non‐HFE‐related HH	2b	*HAMP*	Hepcidin	AR	Juvenile	#602390
Non‐HFE‐related HH	3	*TFR2*	TfR2	AR	Classic	#604720
Non‐HFE‐related HH	4	*SLC40A1*	Ferroportin	AD	Atypical	#606069
Digenic		*HFE* and/or non‐*HFE*		AR	Classic	
Molecularly undefined		Molecular characterization not available				

Abbreviations: AD, autosomal dominant; AR, autosomal dominant; HH, hereditary hemochromatosis; OMIM, online mendelian inheritance in man.

In case of absence of homozygous HFE mutations, ACG and EASL guidelines suggest eliminating other causes of hyperferritinemia and quantifying iron load (MRI imaging, liver biopsy) before investigating non‐HFE hemochromatosis [7, 38]. Non‐consensus diagnostic criteria for HAMP‐ and HJV‐related hemochromatosis have been published; however, for hemochromatosis patients who are not p.C282Y homozygotes, testing for rare variants is recommended [7]. The core gene set for hemochromatosis assessment should include *HFE, HAMP, HJV, TFR2, TF, CP, BMP6*, and *SLC40A1* [39]. Clinical exome or panel gene sequencing may identify more candidate genes, though interpretation is often challenging. Gene‐associated phenotypes vary, and discussions with iron metabolism specialists are recommended [40].

Non‐invasive MRI is not a mandatory part of diagnosis, however, its role in assessing tissue iron concentrations using specific well‐validated sequences has been established. Thus, in patients homozygous for the p.C282Y HFE gene variant with elevated transferrin saturation and hyperferritinemia, MRI is not required for diagnosis, but it is considered helpful in order to determine the extent of iron overload, predicting organ damage [14]. Hepatic MRI R2* may reflect total body iron stores, further guiding therapeutic interventions [14]. EASL guidelines recommend quantifying iron overload in cases of no detection of mutated *HFE* variants, before proceeding to further genetic testing of rarer genetic variants (*HAMP, HJV*) [7]. For those without p.C282Y homozygosity or with other risk factors (e.g., metabolic syndrome, chronic alcohol use, hepatitis C), non‐invasive iron quantification in the liver, spleen, pancreas, and heart may guide diagnosis and management [7, 39].

**FIGURE 3:**
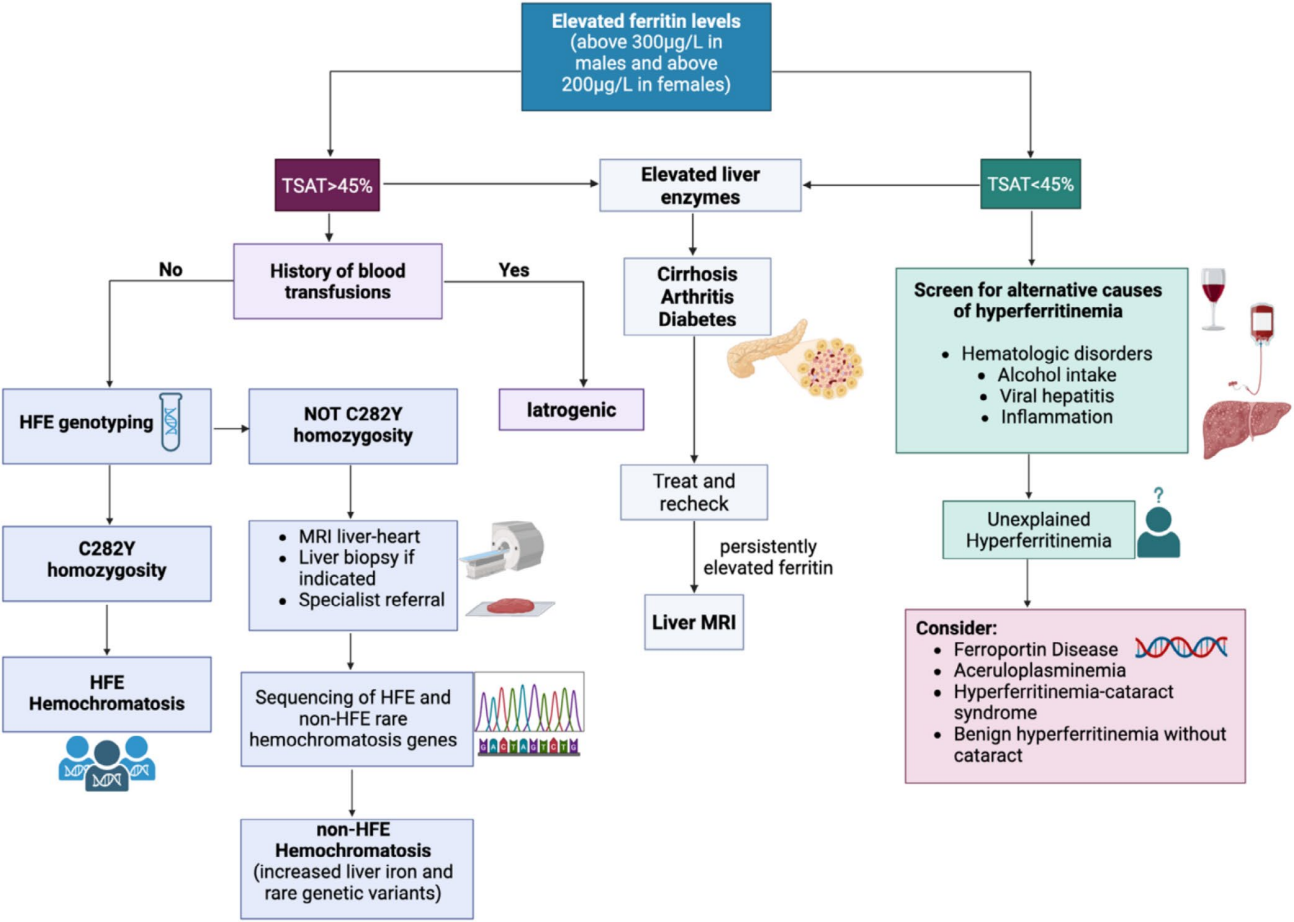
Diagnostic algorithm for iron overload based on EASL guidelines 2022. MRI, magnetic resonance imaging; TSAT, transferrin saturation. The figure was created using BioRender software version 04, License #DF295YLN93.

An analytical algorithm for diagnostic evaluation of iron overload is presented in Figure 3.

## 5 Monitoring

Patients with diseases of iron overload require regular monitoring of serum iron levels, ferritin and transferrin saturation; every 3–6 months during initial treatment and then annually, once levels are normalized [7].

Liver iron concentration (LIC) represents the most reliable indicator of body iron load and is calculated using the formula [Total body iron stores in mg/kg = 10.6 x LIC (in mg/g dry weight)] [20]. Normal LIC values are generally up to 1.8 mg/g dry weight while LIC values up to 7 mg/g dry weight are not generally associated with severe complications [20]. LIC values exceeding 15–20 mg/g are associated with poor prognosis, progression of fibrotic changes and a higher risk for developing hepatocellular carcinoma [41]. The correlation between LIC and extrahepatic iron accumulation is variable and is affected by multiple factors like underlying disease and chelation therapy. In this respect, iron typically accumulates in the liver before affecting the heart, while chelation therapy removes iron from the liver at a quicker pace compared to the heart [42]. As a result, although LIC suggests an increased risk of cardiac iron overload, it does not represent an accurate predictor of myocardial iron levels or related cardiac risks, and myocardial iron can still be present even if LIC is well‐controlled [20]. Established correlation between serum ferritin and iron overload is lacking, making LIC particularly useful when implementing new chelation regimens. At high serum ferritin levels (above 4000 μg/L) the ferritin‐LIC correlation becomes non‐linear, and patients may experience a decrease in LIC without a significant decrease in serum ferritin, especially during the first 6–12 months of treatment [20]. In cases that patients fail to demonstrate a decline in serum ferritin over several months, changes in LIC may help to determine whether their treatment regimen needs adjustment.

Liver biopsy was the standard method for measuring LIC; however, due to its invasiveness and its lack of accuracy, especially in cases of uneven iron distribution, it has been replaced by MRI imaging for LIC measurement [41]. MRI uses magnetic fields to assess proton relaxation in tissues, utilizing methods like T2* and R2 and offering improved accuracy and shorter acquisition times [43]. Discrepancies may arise from calibration differences across facilities, highlighting the need for standardized protocols [43].

Myocardial iron can be estimated using MRI with T2* techniques, which provides useful information critical for monitoring heart‐related risk [7, 44]. Lower T2* values are associated with higher heart failure risks, making regular monitoring crucial for patients undergoing treatment [44].

The aforementioned monitoring techniques, along with their advantages and limitations, are presented in Table 3.

**TABLE 3:** Quantification methods for monitoring iron overload: Advantages and limitations.

Method	Advantages	Limitations
Serum indices‐ferritin	Inexpensive and easy to assess for repeated measurements Correlates with both total body iron stores and clinical outcomes	Non‐linear response to iron load at high levels No decrease does not exclude response to treatment May be influenced by non‐related to iron overload conditions (e.g., inflammation, liver disease)
Serum indices‐TSAT	Inexpensive and easy to assess for repeated measurements Reflects NTBI/LPI pool (over 70%: NTBI significantly increased)	Not reliable for monitoring secondary iron overload (e.g., regular transfusions) High biological variability May be influenced by non‐related to iron overload conditions (e.g., inflammation, liver disease)
MRI (liver and heart)	Non‐invasive Safe T2/T2* linearly related to LIC Measurement of morphological and functional parameters Multi‐organ evaluation	Costly Not always available Indirect quantification of iron overload
Liver biopsy	Direct quantification of iron overload Evaluation of liver histology	Invasive Risk of complications Inadequate sample size of iron distribution

Abbreviations: LIC, liver iron concentration; LPI, labile plasma iron; MRI, magnetic resonance imaging; NTBI, non‐transferrin‐bound iron.

Cardiac evaluation with echocardiogram every 1–2 years is recommended if cardiac involvement is present [7]. In some types of inherited hemochromatosis (HFE‐related, TFR2‐related) that present with an intermediate phenotype in terms of time of onset and clinical features, the need for cardiac MRI at diagnosis should be based on patient's age, disease severity and response to treatment [7]. In contrast, given the high incidence of cardiac involvement in juvenile type of hemochromatosis (HJV‐related) all patients should undergo comprehensive evaluation at diagnosis, with follow‐up tailored to the severity, stage of the disease, clinical manifestations, effectiveness of iron removal, and adherence to therapy [7, 45]. Consultation with a cardiologist is advised if there are signs of potential cardiac involvement. Patients experiencing heart failure and arrhythmias, as well as individuals at risk for iron overload cardiomyopathy (due to genetics, e.g., *HJV* mutations, or medical history) should undergo echocardiograms every 1–2 years to detect any abnormal ventricular diastolic function or decreased peak systolic tissue velocity [7]. Individuals showing higher iron content on cardiac MRI or cardiac biopsy may require more frequent screenings [7].

Regular clinical evaluation to assess symptoms and treatment response is also recommended. Blood glucose testing is recommended annually, in order to monitor diabetes, while serum and urine renal function indices need to be monitored for any sign of deterioration, in order to prevent acute renal failure due to hemosiderin accumulation in renal tubular epithelial cells [7, 46]. MRI has been proven useful in detecting correlation between imaging findings and biochemical markers of pituitary damage, which is of great importance in the management of the disease, considering the impact of iron overload on the endocrine system and that pathological conditions (e.g., diabetes) may only present symptoms after significant damage [20, 42]. Further endocrine evaluations should be guided by clinical symptoms and include assessments for sex hormone concentrations, and occasionally thyroid, adrenal, and parathyroid function [7]. For cirrhotic patients, who have an estimated 20‐ to 200‐fold risk for hepatocellular carcinoma, it is recommended to be screened every 6–12 months by measuring serum alpha‐fetoprotein in combination with liver ultrasonography and/or liver MRI [7]. While some HFE mutations have been linked to extra‐hepatic malignancies, there are no current adjusted screening guidelines recommended for these patients [47].

## 6 Discussion and Future Directions

Iron overload, regardless if it is genetic or acquired, represents a continuously evolving field of hematology. Recent advances in understanding the pathophysiologic mechanisms and molecular basis of iron metabolism have led to the new hemochromatosis classification, based on molecular subtypes, by the International Society for the Study of Iron in Biology and Medicine (BIOIRON Society) [8]. At the same time, guidelines in the management of inherited and acquired disorders of iron overload have been updated during the last 5 years [7, 20]. According to the current recommendations, diagnosis of iron overload involves several key parameters. Elevated serum ferritin levels (above 300 μg/L in males and above 200 μg/L in females) suggest excess iron accumulation. It is important to rule out inflammation, infections, and liver disorders affecting ferritin levels to proceed in further diagnostic algorithms and therapeutic interventions. In cases of high serum ferritin and a TSAT over 45%, in the absence of a secondary cause of iron overload, genetic testing for iron overload‐related genes is recommended [7]. MRI is used to assess iron in parenchymal organs (heart, liver) and to monitor disease progression and response to treatment, since serum ferritin does not fully reflect total body iron [7, 20]. Nowadays, severe iron overload, characterized by cirrhotic liver, multiple endocrine dysfunction, and severe heart failure is rarely seen in clinical practice [48]. This can be attributed to the increased clinical awareness of the diseases associated with iron overload and to the routine assessment of iron biomarkers, particularly serum ferritin and transferrin saturation [8].

NTBI is considered the primary route for iron distribution to the liver and other parenchymal organs in patients with iron overload disorders, thus its increasing levels might be expected to correlate with increasing tissue damage [49]. Studies have demonstrated correlations between NTBI or LPI and cardiac iron markers or response to chelation. Nevertheless, they have not been shown to be reliable predictors of cardiac disease for routine clinical use [20]. Further investigation is needed for specifying the utility of NTBI in guiding routine diagnostic evaluation, prognosis and treatment. Except NTBI, other non‐invasive techniques have been developed in recent years in order to identify disease progression and advanced clinical manifestations (e.g., fibrosis). The role of transient elastography in monitoring iron overload disorders has also not been fully identified yet. The 2019 ACG guidelines did not routinely recommend transient elastography for fibrosis assessment [38], however, the latest EASL guidelines advise using transient elastography at diagnosis to evaluate liver fibrosis and guide treatment and follow‐up in patients with hemochromatosis [7]. They indicate that liver stiffness of 6.4 kPa or less rules out advanced fibrosis, liver stiffness between 6.4 and 12 kPa indicates the need for liver biopsy to confirm the absence of advanced fibrosis, and liver stiffness over 12 kPa is also an indication for liver biopsy, particularly in cases where ferritin is not elevated, transaminase levels are normal, and hepatomegaly is absent [7]. Further research is needed for validating these cut‐off values for diagnosis fibrosis in iron overload disorders.

Another important research area is the utility of genetic testing through population screening. The UK Biobank Study has reshaped our understanding of mortality and morbidity in individuals with homozygous C282Y genotypes discovered through population screening [14]. The C282Y genetic test could be beneficial for young (above 18 years of age) white men with this genotype to detect early disease and begin therapeutic interventions in order to prevent cirrhosis and hepatocellular carcinoma [50]. Findings from this study emphasize the importance of targeted screening for hemochromatosis risk individuals. Initially, only 12.1% of men and 3.4% of women had a known hemochromatosis diagnosis, percentages which increased to 21.7% and 9.8% after genetic testing, revealing significant gaps in detection and awareness [50]. The study also revealed increased mortality and morbidity in both genders [50]. All these issues may partially be resolved or prevented with timely diagnosis and iron reduction therapy at an early age.

Overall, the prognosis for patients with inherited or acquired diseases of iron overload may be enhanced using traditional (serum ferritin, LIC) and non‐traditional (liver stiffness) biomarkers. The monitoring techniques analyzed in detail in this article assist in evaluating iron accumulation in various organs, guiding treatment strategies. Genetic testing, when indicated, provides the diagnosis according to the updated hemochromatosis classification, aiding in the development of personalized therapies.

## 7 Conclusions

Systemic iron overload represents a significant clinical challenge, which requires a thorough comprehension of its pathophysiology and an accurate approach to its clinical and radiological diagnosis and monitoring. Prompt identification of the cause underlying chronic hemosiderosis and proper severity assessment are crucial for treatment guidance and for averting serious complications in vital organs. The advancements of genetic testing and imaging techniques, alongside an improved understanding of underlying mechanisms, have successfully enhanced clinical outcomes for affected patients.

## Author Contributions

E.C., P.D., and A.K. designed and directed the manuscript; E.C., P.D., and K.B. drafted the manuscript. A.K. revised the manuscript. All authors read and approved the final manuscript for publication.

## Funding

The authors have nothing to report.

## Conflicts of Interest

The authors declare no conflicts of interest.

## Data Availability

The data that support the findings of this study are available on request from the corresponding author. The data are not publicly available due to privacy or ethical restrictions.
